# Type 1 conventional dendritic cells regulate innate immunity during fungal pneumonia

**DOI:** 10.1128/mbio.02564-25

**Published:** 2025-09-22

**Authors:** Pari M. Dhruva, Thomas E. Draper, Camille A. Chiller, MaryJane Jones, Chad Steele

**Affiliations:** 1 Department of Microbiology and Immunology, School of Medicine, Tulane University 5783, New Orleans, Louisiana, USA; Instituto Carlos Chagas, Curitiba, Brazil

**Keywords:** fungal, lung, innate immunity

## Abstract

*Aspergillus fumigatus* is a ubiquitous mold that is the primary etiological agent of invasive pulmonary aspergillosis (IPA), a severe lung infection in immunocompromised patients with extreme mortality. Innate immunity is critical for controlling the severity of IPA. Regarding dendritic cells (DCs), studies have shown protective roles for monocyte-derived DCs and plasmacytoid DCs during IPA. Here, we examined the role of type 1 conventional dendritic cells (cDC1s) during *A. fumigatus* lung infection. We first show that global depletion of DCs resulted in impaired lung clearance of *A. fumigatus*, indicating a protective role for DCs, as expected. Unexpectedly, however, mice lacking cDC1s (*Batf3*−/−) cleared *A. fumigatus* more efficiently than wild-type C57BL/6 control mice. Enhanced fungal clearance was also observed in two additional cDC1-deficient strains, Cd11cCre/Irf8flox/flox mice and interferon regulatory factor 8 +32−/− mice. *Batf3*−/− mice displayed an enhanced type 17 immune response, which we have shown to be required for optimal immunity during IPA. Moreover, elevated type 17 responses correlated with differential production of IL-33 (decreased in *Batf3*−/−) and PGE2 (increased in *Batf3*−/−), an axis we have reported to promote type 17 responses during IPA. We further show that alveolar macrophages from *Batf3*−/− mice had increased fungicidal activity against *A. fumigatus*. In contrast, alveolar macrophages cultured in the presence of bone marrow-derived cDC1s had lower fungicidal activity compared to alveolar macrophages cultured alone, suggesting that cDC1s restrict alveolar macrophage function. Taken together, our data identify cDC1s as a regulator of innate immune responsiveness during IPA.

**IMPORTANCE:**

Fungal infections have increased at an alarming rate as a result of increased usage of immunosuppressive therapies, growing resistance to antifungal drugs, and global warming. This recently prompted the World Health Organization to publish the first-ever fungal priority pathogens list, which focused on 19 organisms, ultimately deeming 4 pathogens of critical importance based on perceived public health importance. Among these four was the opportunistic mold *Aspergillus fumigatus*, the etiological agent of the most lethal fungal infection known to humans, IPA. Innate immunity is paramount for controlling IPA with protective roles identified for multiple myeloid cell types. In the current report, employing three complementary animal models, we show that cDC1s hinder the clearance of *A. fumigatus* from the lung. We further identified specific responses that are regulated by cDC1s. Overall, our study uncovers a new mechanism of immune regulation during IPA.

## INTRODUCTION

Severe fungal infections have emerged as a significant global health concern over the past decade, with an estimated 6 million life-threatening infections worldwide causing more than 2.5 million deaths annually (1). This worrisome trend can be attributed, in part, to rising temperatures and the increasing drug resistance toward available antifungals, especially with *Candida* and *Aspergillus* spp. (2–4). Recent estimates from the Centers for Disease Control and Prevention indicate the economic burden of fungal disease in the USA annually is nearly $20 billion in medical costs, absenteeism, and death (5). Among these fungal infections with the highest mortality is the ubiquitous mold *Aspergillus fumigatus*, the primary case of a fungal pneumonia termed invasive pulmonary aspergillosis (IPA) (6, 7). IPA predominantly affects immunocompromised patients, including recipients of stem cell or solid organ transplants and individuals in intensive care units (8). IPA also occurs in over 2 million people in the context of chronic obstructive pulmonary disease with over 85% mortality rate (1).

Innate immunity serves as a crucial defense barrier against IPA (9). While macrophages and neutrophils have long been recognized as key components of the immune defense against *A. fumigatus* in the lung (10, 11), the role of dendritic cells (DCs) in IPA is less studied. Early reports indicated that lung CD11c + DCs utilized the mannose receptor and C-type lectins that recognize galactomannan to identify *A. fumigatus* conidia, whereas CR3 and FcγR were utilized to recognize hyphae (12). DC-SIGN is an additional mannan/mannose-specific receptor employed by DCs to recognize *A. fumigatus* (13). Common chemokine receptors expressed by DCs during IPA include CCR2 (14–16), CCR6 (17), and CCR7 (18). CCR2 is most often expressed by monocyte-derived DCs (mo-DCs) (14–16), whereas CCR7 often marks DCs migrating to the secondary lymphoid tissues (18). Curiously, however, mice deficient in CCR7 are more resistant to IPA due to increased numbers of DCs in the lung that had a more activated and mature phenotype (18).

DCs are typically divided into three main types at steady state: plasmacytoid dendritic cells (pDCs), type 1 conventional dendritic cells (cDC1s), and type 2 conventional dendritic cells (cDC2) (19, 20). However, during inflammation, mo-DCs may arise (14, 16) as well as a cDC2 subset that acquires a hybrid inflammatory-cDC2 (inf-cDC2) phenotype that shares phenotype, gene expression, and function with cDC1s and monocyte-derived cells (20). Some studies have investigated the role of various DC subsets during IPA, which have identified protective roles for pDCs (15, 21–23) and mo-DCs (14–16). In the current report, we sought to define the role of the cDC1 subset in lung immune responses during IPA.

## MATERIALS AND METHODS

### Mice

Male and female C57BL/6 mice, 6–8 weeks of age, were obtained from The Jackson Laboratory (Bangor, ME). *Batf3*−/− mice (Jackson Labs stock #013755) (24) were a gift from Dr. James McLachlan, Tulane University. Interferon regulatory factor 8 (Irf8) +32−/− mice, which have a deletion in a critical enhancer region of the Irf8 gene (25), were purchased from Jackson Labs (stock #032744). Cd11c Cre mice (26) (Jackson Labs, stock #008068) were crossed with Irf8 floxed mice (27) (Jackson Labs, stock #014175) to delete Irf8 from Cd11c+ cells. CD11c-DTR mice were purchased from Jackson Labs (stock # 004509). CD11c-DTR mice were administered 100 ng of diphtheria toxin (DT; List Labs, cat. #150, lot #15045A1) intraperitoneally 12 h prior to infection to deplete DCs. As DT has been reported to have adverse effects in normal (28) and Cd11c-DTR (29, 30) mice, to account for any off-target effects of DT, WT C57BL/6 mice were treated with DT as a control.

### Preparation of *A. fumigatus*, *in vivo* challenge, and lung fungal burden assessment

*A. fumigatus* isolate 13073 (American Type Culture Collection, Manassas, VA) was maintained on potato dextrose agar for 5–7 days at 37°C. Conidia were harvested by washing the culture flask with 50 mL of sterile phosphate-buffered saline (PBS) supplemented with 0.1% Tween 20. The conidia were then passed through a sterile 40 µm nylon membrane to remove hyphal fragments and enumerated on a hemocytometer. For *in vivo* challenge, mice were lightly anesthetized with isoflurane and administered 7 × 10^7^ *A. fumigatus* conidia in a volume of 50 µL intratracheally as we have previously reported (31–33). Note that as we are examining the role of cDC1s in the innate immune response to *A. fumigatus*, we did not employ immunosuppression. For lung fungal burden analysis, the left lungs were collected at 24 h post-exposure and homogenized in 1 mL of PBS. Total RNA was extracted from 0.1 mL of unclarified lung homogenate using the MasterPure yeast RNA purification kit (Epicenter Biotechnologies, Madison, WI), which includes a DNAse treatment step to eliminate genomic DNA as we have previously reported (31–34). Total RNA was also extracted from serial 1:10 dilutions of live *A. fumigatus* conidia (10^1^–10^9^) and DNase treated to form a standard curve. Lung *A. fumigatus* burden was analyzed with real-time PCR measurement of the *A. fumigatus* 18S rRNA (35) and quantified using a standard curve of *A. fumigatus* conidia as we have extensively reported (31–34).

### Lung cell isolation and culture and inflammatory mediator analysis

For lung cell isolation, the lungs were collected and minced in Iscove’s Modified Dulbecco’s Medium (IMDM) media (MilliporeSigma) supplemented with 1% penicillin-streptomycin-glutamine (Mediatech), 10% heat-inactivated fetal bovine serum (Invitrogen), and 0.4  mg/mL polymyxin B (Thermo Fisher Scientific), followed by incubation for 60 min with tissue culture grade type IV collagenase (1 mg/mL, MilliporeSigma) in a 37°C orbital shaker at 100 rpm. The cell suspension was filtered through sterile 70 and 40 µm nylon filters, and red blood cells were lysed with ACK buffer (Lonza) to create single-cell preparations. One million cells in a volume of 200 µL were cultured overnight with 1 million *A. fumigatus* conidia (1:1), followed by collection and clarification of supernatants. Supernatants were analyzed for protein levels of 32 cytokines and chemokines using a Milliplex multiplex suspension cytokine array (Millipore) according to the manufacturer’s instructions. The data were analyzed using Bio-Plex Manager software (Bio-Rad Laboratories). IL-33, PGE2 analysis, and IL-22 levels were quantified by enzyme-linked immunosorbent assay (R&D Systems).

### Flow cytometry

Lung cells were isolated as described above. Cells were washed and Fc receptors were blocked with Mouse BD Fc Block (BD Biosciences, San Diego, CA) at 4°C for 20 min. Thereafter, cells were stained with a single-color LIVE/DEAD Fixable Dead Cell Stain (Invitrogen) followed by labeling with specific immune cell surface markers. The following staining parameters were employed: eosinophils as CD45+, CD11b+ Siglec F+ Ly-6G−; neutrophils as CD45+ CD11b+ Ly6G+; inflammatory monocytes as CD45+, CD11b+ Ly6C+ CCR2+; alveolar macrophages as CD45+, CD11c+, CD11b−, F4/80+, Ly6C−; interstitial macrophages as CD45+, CD11c−, CD11b+, F4/80+; and dendritic cells gated as CD45+, CD11c+, Ly6C+, MHC II+ (all antibodies purchased from Biolegend, eBiosciences and BD Biosciences). Dendritic cell subsets were identified through a series of markers, beginning with CD45+, CD11C+, MHC II+, and CD26+ (20, 36). From this population, further subsets were distinguished: cDC1s were characterized by Xcr1+, Sirpa−, and CD103+ expression; cDC2s were identified as Xcr1−, Sirpa+, CD64−, FceR1a−, and Cd11b+, while inflammatory cDC2s showed Xcr1−, Sirpa+, CD64+, and FceR1a+ expression. Monocyte-derived dendritic cells were defined by CD45+, CD11C+, IA IE+, CD26−, CD88+, Sirpa+, Xcr1−, CD64+, and FceR1a+ markers. Lastly, plasmacytoid dendritic cells were gated based on CD11c+, CD11b−, Ly6C+, Ly6G−, BST2+, and B220+ expression (37). The supplemental data include the gating strategy for each DC subset (Fig. S1) and the antibodies employed (Table S1).

### Assessment of *in vitro A. fumigatus* killing by alveolar macrophages and neutrophils

Bronchoalveolar lavage was performed on C57BL/6 and *Batf3*−/− mice to isolate alveolar macrophages as previously described (31). For *A. fumigatus* killing *in vitro*, macrophages (1 × 10^5^) were cocultured at a 1:1 effector-to-target (E:T) ratio with live *A. fumigatus* resting conidia for 24 h followed by RNA isolation with the MasterPure yeast RNA purification kit and real-time PCR assessment as described above. Controls included *A. fumigatus* resting conidia cultured in the absence of macrophages for 24 h. Bone marrow (BM) cells were collected from the femurs and tibiae of C57BL/6 and *Batf3*−/− mice by flushing the opened bones with IMDM (Invitrogen). Neutrophils were isolated from bone marrow cell pellet using Neutrophil Isolation Kit (Miltenyi). For *A. fumigatus* killing *in vitro*, neutrophils (1 × 10^6^) were cocultured at a 1:1 E:T ratio with live A. fumigatus resting conidia for 24 h, followed by RNA isolation with the MasterPure Yeast RNA Purification Kit and real-time PCR assessment as described above. Controls included *A. fumigatus* resting conidia cultured in the absence of neutrophils for 24 h.

### cDC1 generation and coculture with alveolar macrophages

Bone marrow cells were isolated from naive C57BL/6 mice. Cells were cultured in the presence of continuous FLT3 (200 ng/mL) and granulocyte-macrophage colony-stimulating factor (5 ng/mL); every 5 days, non-adherent and loosely adherent cells were collected and replated over the course of 15 days, a protocol that generates basic leucine zipper ATF-like transcription factor 3 (*BATF3*) and IRF8-dependent CD103+cDC1 s (38). Alveolar macrophages were isolated as described above. For A. fumigatus killing *in vitro*, bone marrow-derived cDC1s (0.5 × 10^5^) and alveolar macrophages (0.5 × 10^5^) were cultured separately and together at a 1:1 effector to target ratio with live *A. fumigatus* conidia for 24 h. Fungal killing assessed by real-time PCR was conducted as described above.

### Histology

The left lungs were collected and fixed in 4% formalin. The fixed lungs were paraffin embedded and then processed and stained by GNO Histology Consultants (New Orleans, LA). Imaging was performed using a Swift Optical Instruments M10T-P Trinocular LED Microscope equipped with a Motic Moticam 5 + 5 megapixel digital camera. In specific experiments, *A. fumigatus* organisms were quantified in 40 random high-power fields (40×) from multiple WT and *Batf3*−/− mice.

### Statistics

Data were analyzed using GraphPad Prism version 10.3.1 statistical software. Comparisons between groups when data were normally distributed were made with the Student’s *t*-test. Significance was accepted at a value of *P* < 0.05.

## RESULTS

### Global depletion of DCs results in impaired lung clearance of *Aspergillus fumigatus*

**Fig 1:**
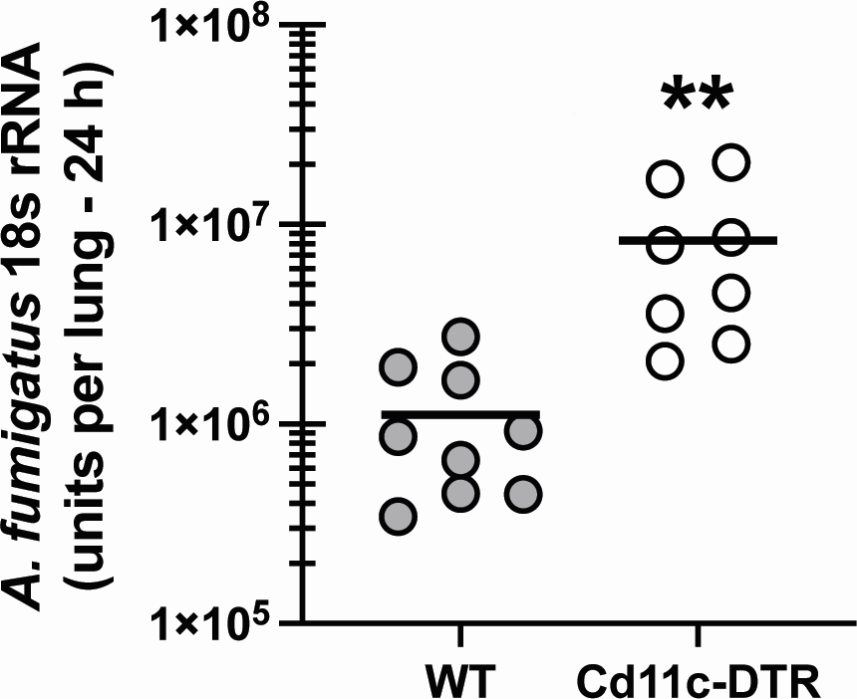
Global depletion of DCs results in impaired lung clearance of *Aspergillus fumigatus*. C57BL/6 wild-type (WT) and CD11c-DTR transgenic mice were challenged intratracheally with 7 × 10^7^ *A. fumigatus* conidia. Lung fungal burden at 24 h post-challenge was assessed by real-time PCR analysis of *A. fumigatus* 18S rRNA levels. The figure illustrates cumulative data from two independent studies (*n* = 4–5 mice per group per study). Each symbol represents an individual mouse. Line within a given group represents the mean. **P < 0.01 (unpaired two-tailed Student’s *t*-test).

Deficiency in pDCs results in enhanced mortality with IPA (60%–75% by day 5 post-challenge) (21). Although data appear to also indicate a role for mo-DCs in IPA lung defense, a closer examination of these reports indicates that functions/mechanisms attributed to mo-DCs may also be attributed to inflammatory monocytes (14–16). Mice deficient in IRF4-dependent CD24+CD11b+ DCs (now called cDC2s) have been reported to have a defect in *A. fumigatus* lung clearance (39), although this was analyzed at an unusually late time point, 7 days post-challenge, which detected very little organism in the lung (i.e., control vs experimental, ~50 CFU/lung vs ~250 CFU/lung). This study also employed a low inoculum (2 × 10^7^) given intranasally, and the control for Cd11c Cre/Irf4 floxed mice was normal BL/6 mice rather than the Cd11c Cre or Irf4 floxed mice (39). To confirm that DCs played a role in *A. fumigatus* lung clearance with a standard inoculum (7 × 10^7^ conidia) administered via a standard route (intratracheally) and at a relevant innate immune time point (24 h post-challenge), we employed CD11c-DTR mice to deplete CD11c+ DCs via diphtheria toxin treatment. Results in Fig. 1 show that mice depleted of CD11c+ DCs have increased lung fungal burden at 24 h post-infection. Thus, DCs are important early antifungal effector cells against *A. fumigatus*.

### cDC1-deficient mice display enhanced *Aspergillus fumigatus* lung clearance

**Fig 2:**
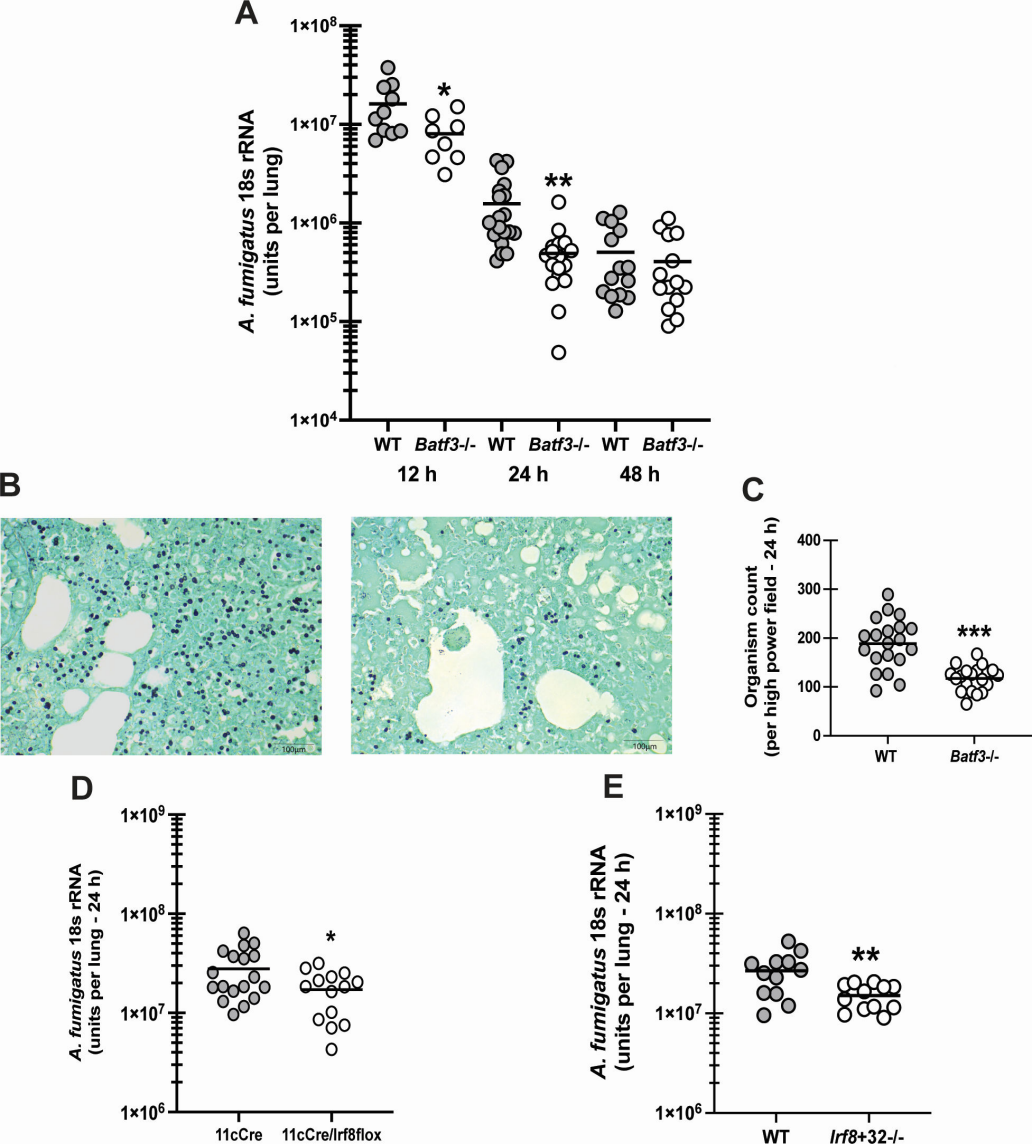
cDC1-deficient mice display enhanced *Aspergillus fumigatus* lung clearance. C57BL/6 wild-type (WT) and cDC1-deficient mice (*Batf3*−/−) mice were challenged intratracheally with 7 × 10^7^ *A. fumigatus* conidia. (**A**) Lung fungal burden at 12, 24, and 48 h post-challenge was assessed by real-time PCR analysis of *A. fumigatus* 18S rRNA levels. The figure illustrates cumulative data from two to three independent studies (*n* = 4–5 mice per group per study). Each symbol represents an individual mouse. Line within a given group represents the mean. (**B**) Representative GMS stained lung sections from WT mice (left) and *Batf3*−/− mice (right) 24 h post-challenge. Original magnification ×40. Scale bar equals 100 µm. (**C**) *A. fumigatus* organisms were quantified in 40 random high-power fields (40×) from GMS-stained lung sections of WT and *Batf3*−/− mice. The figure illustrates cumulative data from two independent studies (*n* = 4 mice per group per study). (**D**) Cd11c Cre and Cd11c Cre/Irf8 floxed mice were challenged intratracheally with 7 × 10^7^ *A. fumigatus* conidia. Lung fungal burden 24 h post-challenge was assessed by real-time PCR analysis of *A. fumigatus* 18S rRNA levels. The figure illustrates cumulative data from three independent studies (*n* = 4–5 mice per group per study). Each symbol represents an individual mouse. Line within a given group represents the mean. (**E**) C57BL/6 WT and *Irf8 +32*−/− mice were challenged intratracheally with 7 × 10^7^ *A. fumigatus* conidia. Lung fungal burden 24 h post-challenge was assessed by real-time PCR analysis of *A. fumigatu*s 18S rRNA levels. The figure illustrates cumulative data from two independent studies (*n* = 6 mice per group per study). Each symbol represents an individual mouse. Line within a given group represents the mean. For all graphs: *P < 0.05, **P < 0.01, ***P < 0.001 (unpaired two-tailed Student’s *t*-test).

As previously discussed, pDCs, mo-DCs, and cDC2s are reported to play a role in the lung immune response during IPA. Indeed, global depletion of DCs in Fig. 1 supports their requirement for fungal clearance. As DC subsets may have different functions, we questioned whether cDC1s were similarly required for lung defense during IPA. *BATF3* is a transcription factor that is responsible for the development of CD103+cDC1 s (24). We therefore exposed *Batf3*−/− mice to *A. fumigatus* and measured lung fungal burden at 12, 24, and 48 h post infection. Unexpectedly, results in Fig. 2A show that *Batf3*−/− mice have enhanced clearance of *A. fumigatus* as early as 12 h after challenge, which is even more pronounced 24 h after exposure. However, by 48 h, the level of *A. fumigatus* in the lung is similar between WT and *Batf3*−/− mice. Representative GMS-stained lung tissue from WT BL/6 (Fig. 2B, left) and *Batf3*−/− mice (Fig. 2B, right) 24 h post-infection supports higher *A. fumigatus* levels in the former. In addition, enumeration of *A. fumigatus* organisms in random high-power fields demonstrated ~40% fewer organisms in *Batf3*−/− mice (Fig. 2C). IRF8 is an additional transcription factor that regulates cDC1 development (40). Therefore, to further examine the role of cDC1s, we challenged Cd11cCre/Irf8flox/flox mice, in which Irf8 is deleted in CD11c+ cells, leading to reduced cDC1 numbers (41). Similar to *Batf3*−/− mice, Cd11cCre/Irf8flox/flox mice had significantly lower *A. fumigatus* lung burden at 24 h compared to Cd11cCre control mice (Fig. 2D). cDC1s were reduced by more than 500-fold in Cd11cCre/Irf8flox/flox mice (5.8 × 10^3^ ± 7.2 × 10^2^ vs 1 × 10^1^ ± Cd11cCre vs Cd11cCre/Irf8flox/flox, respectively; *P* = 0.0002). Finally, to provide additional evidence that the absence of cDC1s improved *A. fumigatus* lung clearance, we examined *Irf8 +32*−/− mice in which CRISPR/Cas9- was employed to delete an enhancer located in the super-enhancer region of Irf8 (25). Here again, similar to *Batf3*−/− and Cd11cCre/Irf8flox/flox mice, *Irf8 +32*−/− mice also had significantly lower *A. fumigatus* lung burden 24 h post-challenge (Fig. 2E). cDC1s were more than 36-fold lower in *Irf8 +32*−/− mice (1.03 × 10^4^ ± 1.1 × 10^3^ vs 2.9 × 10^2^ ± 4 × 10^1^, WT vs *Irf8 +32*−/− mice, respectively; *P* < 0.0001). Thus, the presence of cDC1 regulates the magnitude of fungal clearance early after *A. fumigatus* challenge.

### The absence of cDC1s results in specific lung cellular changes during fungal pneumonia

**Fig 3:**
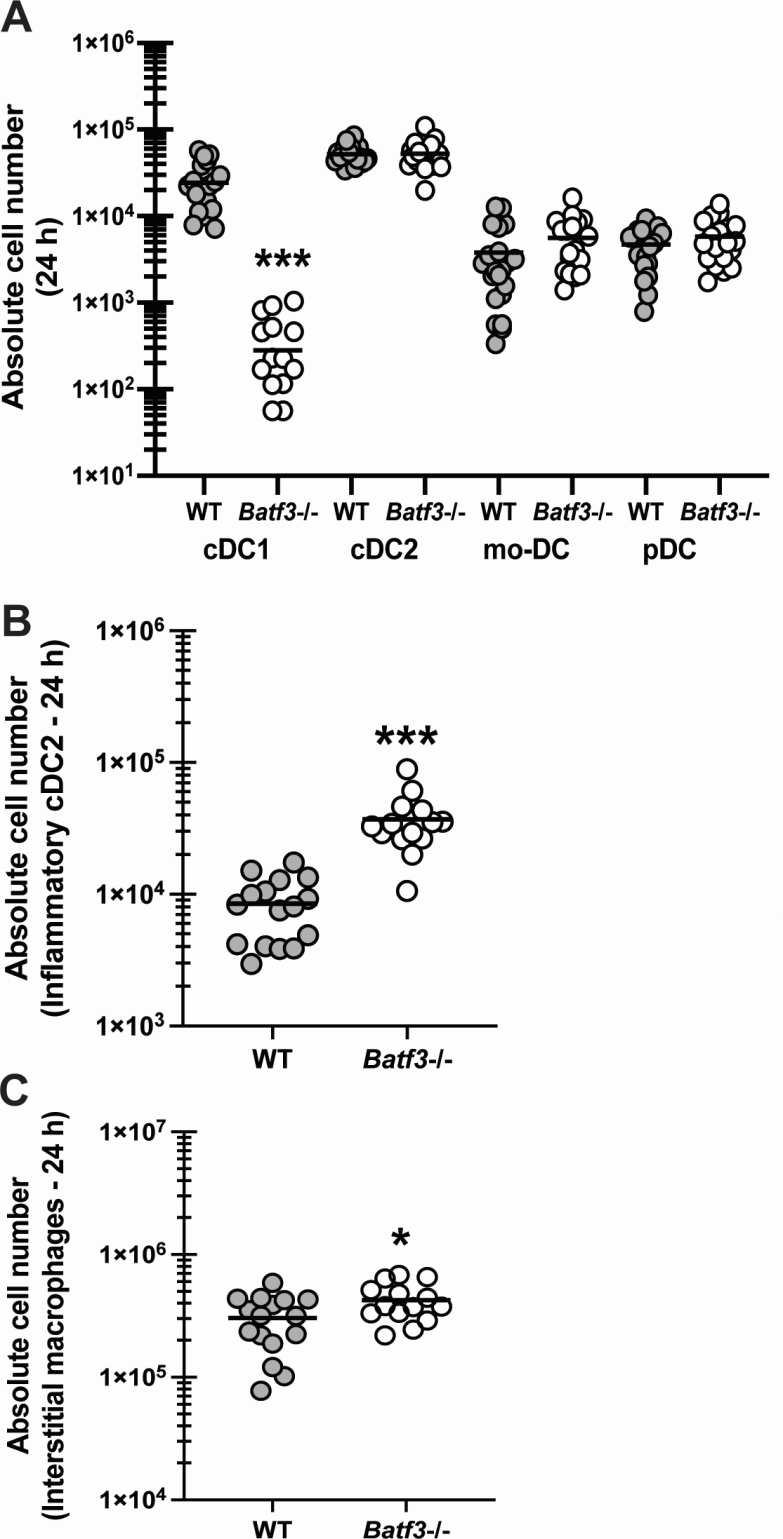
The absence of cDC1s results in specific lung cellular changes during fungal pneumonia. C57BL/6 wild-type (WT) and cDC1-deficient (*Batf3*−/−) mice were challenged intratracheally with 7 × 10^7^ *A. fumigatus* conidia. At 24 h post-challenge, lungs were collected and enzymatically digested. (**A**) cDC1, cDC2, mo-DC, and pDC subsets, (**B**) inflammatory cDC2s, and (**C**) interstitial macrophages were quantified by flow cytometry (markers listed in Materials and Methods). The figures represent cumulative data from three independent studies (*n* = 5–6 mice per group per study). Each symbol represents an individual mouse. The line within a given group represents the mean. For all graphs: *P < 0.05, *** represent a *P* < 0.001, respectively (unpaired two-tailed Student’s *t*-test).

To understand the extent by which cDC1 deficiency modulated innate cell populations, we first examined DC subsets in *Batf3*−/− mice 24 h post-infection. Results in Fig. 3A confirmed the near absence of the cDC1 subset in *Batf3*−/− mice (~85-fold reduction). In contrast, we did not observe any differences in the levels of cDC2s, mo-DCs, or pDCs between WT and *Batf3*−/− mice (Fig. 3A). The gating strategy for each DC subset is provided in Fig. S1. A previous study examining the murine lung during viral infection employed stringent flow cytometric analysis coupled with bulk and single-cell RNA-seq analysis to characterize DC subsets (8). A major finding of this study was that the inf-cDC2 phenotype shared phenotypes, gene expressions, and functions with cDC1s and monocyte-derived cells. In contrast to the previous four DC subsets, we observed significantly increased levels of inf-cDC2s in *Batf3*−/− mice 24 h post-infection (Fig. 3B). Regarding other innate cell populations, we did not see differences in neutrophils, alveolar macrophages, eosinophils, or inflammatory monocytes between WT and *Batf3*−/− mice (data not shown), although we did observe an increase in interstitial macrophages (Fig. 3C). Thus, the absence of cDC1s results in a compensatory increase in the inf-cDC2 subset.

### cDC1 deficiency promotes a hyperinflammatory state during fungal pneumonia

**Fig 4:**
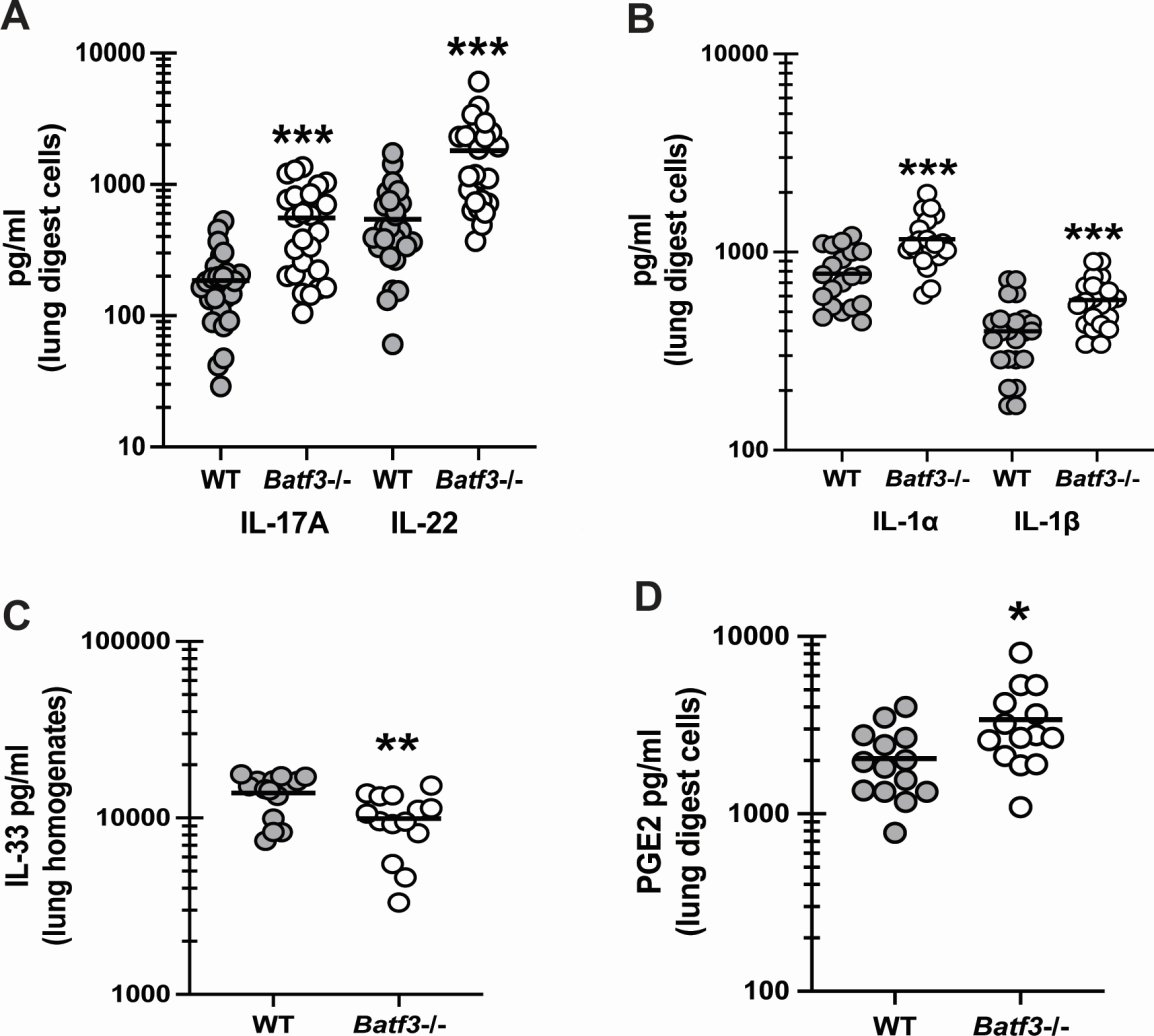
cDC1 deficiency promotes a hyperinflammatory state during fungal pneumonia. C57BL/6 wild-type (WT) and cDC1-deficient (*Batf3*−/−) mice were challenged intratracheally with 7 × 10^7^ *A. fumigatus* conidia. At 24 h post-challenge, lungs were collected, enzymatically digested, and cultured for 24 h. Alternatively, lungs were collected, homogenized, and clarified by centrifugation. (**A**) IL-17A and IL-22, (**B**) IL-1α and IL-1β, (**C**) IL-33, and (**D**) PGE2 levels were quantified in coculture supernatants or clarified lung homogenates by Luminex-based Milliplex assessment or enzyme-linked immunosorbent assay. The figure illustrates cumulative data from three to five independent studies (*n* = 5–7 mice per group, per study). Each symbol represents an individual sample. Line within a given group represents the mean. For all graphs: *P < 0.05, **P < 0.01, ***P < 0.001 (unpaired two-tailed Student’s *t*-test).

We (32, 42, 43) and others (44) have shown the importance of innate (i.e., not T helper) type 17 responses, specifically the production of IL-17A and IL-22, in protection against IPA. Although type 17 responses are widely acknowledged to be critical for neutrophil recruitment, these responses are equally important in generating the epithelial antimicrobial response (45). To this end, we questioned whether the absence of cDC1s affected type 17 responses during *A. fumigatus* lung infection. Data in Fig. 4A demonstrate that both IL-17A and IL-22 were significantly higher in *Batf3*−/− mice. We have previously reported that type 17 responses are highly dependent on IL-1 receptor signaling (33). We found that both IL-1α and IL-1β were significantly elevated in the absence of cDC1s (Fig. 4B). We have also previously reported that mice deficient in the receptor for IL-33 (Il1rl1−/−) had enhanced type 17 responses, which were dependent on PGE2 (33). We have also shown that elevated type 17 responses were increased in mice deficient in acidic mammalian chitinase (AMCase) exposed to *A. fumigatus*, which correlated with IL-33 levels (lower) and PGE2 levels (higher) (46). Data from *Batf3*−/− mice mimicked Il1rl1−/− (IL-33R) (33) and Chia−/− (AMCase) (46) mice in that elevated type 17 responses also correlated with a decrease in IL-33 levels (Fig. 4C) and an increase in PGE2 levels (Fig. 4D). Thus, the absence of cDC1s allows for a more robust protective type 17 response during fungal pneumonia.

### cDC1s modulate innate cell antifungal activity against *A. fumigatus*

**Fig 5:**
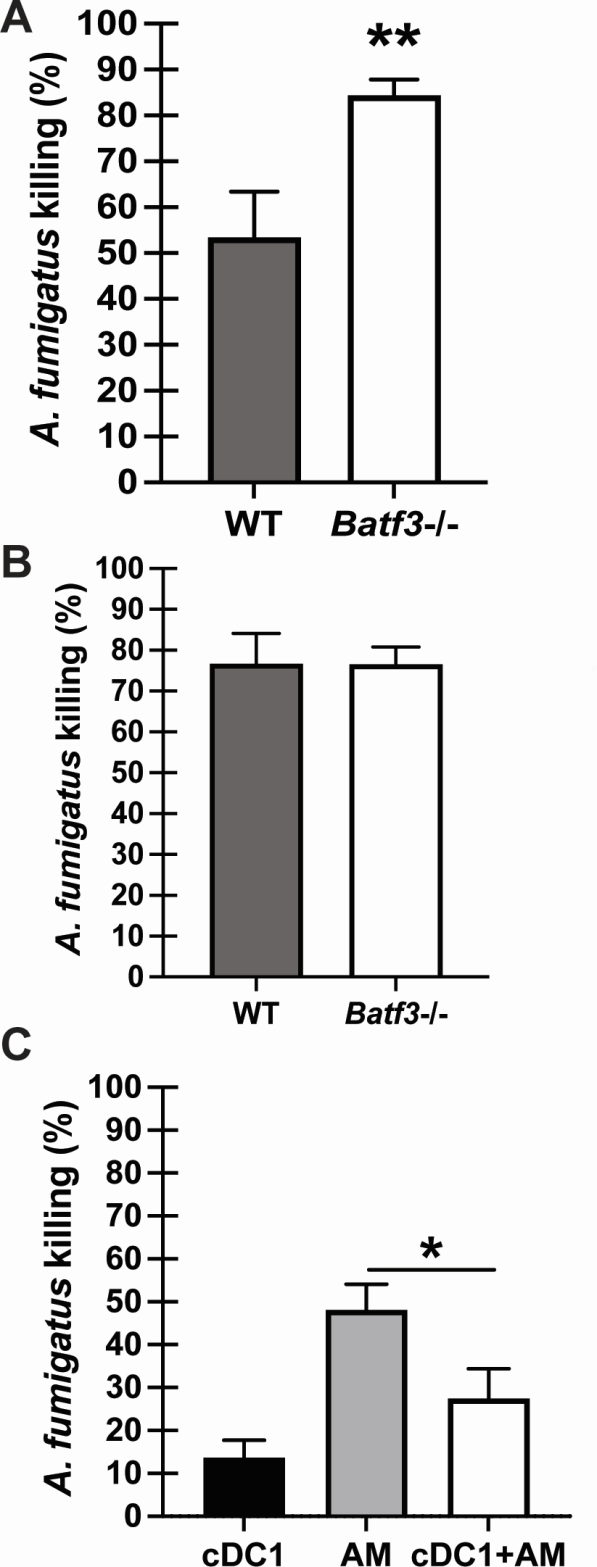
cDC1s modulate innate cell antifungal activity against *A. fumigatus*. (**A**) Alveolar macrophages (AMs) from WT and *Batf3*−/− were plated 1:1 with *A. fumigatus* resting conidia. *A. fumigatus* resting conidia were cultured alone as a control for viability. After 24 h, the total contents of each well were collected, and fungal killing was assessed by real-time PCR analysis of *A. fumigatus* 18S rRNA levels. Data are expressed as mean percentage of fungi killed ± SEM. The figure illustrates cumulative data from three independent studies (*n* = 2–3 mice per group per study). (**B**) C57BL/6 wild-type (WT) and cDC1-deficient (*Batf3*−/−) mice bone marrow-derived neutrophils were plated 1:1 with *A. fumigatus* resting conidia. *A. fumigatus* resting conidia were cultured alone as a control for viability. Fungal killing was assessed as in panel **A**. Data are expressed as mean percentage of fungi killed ± SEM. The figure illustrates cumulative data from two independent studies (*n* = 4–5 mice per group per study). (**C**) Bone marrow-derived cDC1s, AMs, and cDC1s + AMs were plated 1:1 with *A. fumigatus* resting conidia. *A. fumigatus* resting conidia were cultured alone as a control for viability. Fungal killing was assessed as in panel **A**. Data are expressed as mean percentage of fungi killed ± SEM. The figure illustrates cumulative data from two independent studies (*n* = 3–4 mice per group per study). For all graphs: *P < 0.05, **P < 0.01 (unpaired two-tailed Student’s *t*-test).

Alveolar macrophages and neutrophils are primary effector cells in the lung during fungal pneumonia (10, 11). We hypothesize that in the absence of cDC1 signaling, the fungicidal capacity of these innate effector cells may be modulated. Data in Fig. 5A indicate that alveolar macrophages isolated from the lungs of naïve *Batf3*−/− mice displayed enhanced killing of *A. fumigatus* compared to those isolated from naïve WT BL/6 mice. In contrast, neutrophils isolated from the BM of naïve *Batf3*−/− mice demonstrated similar antifungal activity as those from WT BL/6 mice (Fig. 5B). To more rigorously examine enhanced alveolar macrophage killing in the absence of cDC1s, we generated cDC1s from BM (38) and examined their capacity to modulate alveolar macrophage-mediated killing of *A. fumigatus*. BM-derived cDC1s showed a small amount of *A. fumigatus* killing (~15%), which was significantly lower than *A. fumigatus* killing by AMs (~50%) (Fig. 5C). However, when alveolar macrophages were cultured in the presence of cDC1s, *A. fumigatus* killing dropped to ~10% (after removing the 15% killing attributed to cDC1s) (Fig. 5C). Thus, cDC1s negatively affect the ability of alveolar macrophages to kill *A. fumigatus*.

## DISCUSSION

Our initial observation that global depletion of DCs results in impaired *A. fumigatus* lung clearance indicated a protective role for a DC subset. pDCs are known to possess antifungal activity against *A. fumigatus*, and their depletion results in increased susceptibility to IPA (21). Another study has shown that mo-DC-derived and neutrophil-derived CXCL9 and CXCL10 recruit pDCs to the lung during IPA, where they function to enhance neutrophil fungicidal activity (15). Likewise, CCR2+ monocytes and mo-DCs condition the lung to enhance neutrophil function against *A. fumigatus* (14). It has also been shown that deletion of mo-DCs during IPA results in reduced Th1, yet enhanced Th17, responses (47). Neutrophils also appear to regulate DCs in some manner, in that during neutropenia, DC recruitment to the lung is enhanced (48), although these DCs do not efficiently egress to the draining lymph nodes and display a more immature phenotype (49). However, this study reported enhanced recruitment of CD11b+ CD11c+ DCs but not CD103+ DCs (49). Based on thorough characterization of lung DC subsets at baseline and during inflammation (20), CD103+ DCs are cDC1s; however, CD11b+ CD11c+ DCs could be either cDC2s, inf-cDC2s, or mo-DCs. Likewise, studies employing CCR2 depleter mice result in the depletion of not only CCR2+ monocytes and CCR2+ mo-DCs during *A. fumigatus* lung infection (14–16) but also potentially inf-cDC2s, as they depend on CCR2 for migrating to the lungs during viral infection (20). Overall, DC subsets and their function may be more complex than originally thought.

The primary goal of this study was to examine the role of the cDC1 DC subset in fungal pneumonia/IPA. Unexpectedly, we found that *Batf3*−/− mice lacking cDC1s had enhanced early lung clearance of *A. fumigatus*. This observation was surprising, as cDC1-deficient mice have been shown to be susceptible to the lung pathogens vaccinia (50), rhinovirus (51), and *Cryptococcus neoformans* (52), whereas cDC1s do not play a role in influenza (53) or pneumococcal (54) lung infection. To provide further support that cDC1s function in a negative capacity during fungal pneumonia/IPA, we also examine Cd11d Cre/Irf8 floxed mice as well as *Irf8 +32* mice, both of which have defects in cDC1 development (25, 41). In agreement with our data in *Batf3*−/− mice, these additional cDC1-deficient/impaired strains also demonstrated an enhanced ability to clear A*. fumigatus*. Altogether, our data suggest that both protective and non-protective DC subsets are present in the lung during fungal pneumonia/IPA, in that mice globally depleted of DCs have impaired lung clearance of *A. fumigatus*, whereas mice deficient in cDC1s have enhanced clearance.

cDC1s are most widely studied for their ability to present antigens in the form of material internalized from dying tumor or virally infected cells for the induction of protective CD8+ T-cell responses (55). Although antigen-specific cytotoxic CD8 T cells/lines (56–60) and CD8 CAR T cells (61) may be protective after adoptive transfer during *A. fumigatus* lung infection, susceptible experimental strains such as MYD88-deficient mice (62) do not have differences in CD8+ T cells at early (24–48 h) time points after *A. fumigatus* challenge. Moreover, depletion of CD8+ T cells in C57BL/6 mice has no effect on fungal burden 3 days after challenge (59). Therefore, we feel it is unlikely that CD8+ T cells are playing a role in the early time points employed with *Batf3*−/− mice. Less investigated is how cDC1s may function in affecting/modulating the immune response of innate myeloid cells. cDC1s contribute to obesity-associated inflammation by increasing IFN-γ production and inflammatory macrophage accumulation via endocytosis of apoptotic bodies containing self-DNA (63). Another study demonstrated that during *Toxoplasma gondii* exposure, cDC1s coordinated the recruitment of monocytes into the omentum and peritoneum and coordinated recruitment of resident macrophages from the peritoneum into fat-associated lymphoid clusters (64). cDC1s have been shown to express various immunoregulatory genes, such as Tim4, Btla, and Clec9a, during CD8 T-cell responses (65–67). In a model of sepsis, mice deficient in the immunoregulatory gene Btla had decreased mortality in the presence of lower bacterial burden, which correlated with increased activation of multiple myeloid cells, including macrophages (68), suggesting that BLTA functioned to regulate the magnitude of innate cell responsiveness. Other studies have shown that CLEC9A activates the negative regulatory signal SHP-1 to dampen neutrophil-mediated immunopathology during systemic fungal infection (69). We found that alveolar macrophages from *Batf3*−/− mice had enhanced killing of *A. fumigatus in vitro*, suggesting that the presence of cDC1s restricts either the activation or function of alveolar macrophages. This was verified when alveolar macrophages were cultured in the presence of cDC1s, which resulted in significantly abrogated antifungal capacity. Although we do not currently know if cDC1s in the lung express immunoregulatory genes such as Tim4, Btla, or Clec9a, future studies will more thoroughly examine mechanisms of cDC1-mediated regulation of alveolar macrophage function as well as characterize differences in alveolar macrophage activation between normal and *Batf3*−/− mice.

As previously stated, IL-17A and IL-22 are critical effector cytokines for the elimination of *A. fumigatus* from the lung (31, 42–44). In addition to cDC1s regulating alveolar macrophage antifungal activity, we observed that production of IL-17A and IL-22 by lung cells from *Batf3*−/− mice was significantly higher than cells from WT mice, suggesting this is an additional mechanism contributing to the enhanced clearance in the absence of cDC1s. We have previously reported that during IPA, IL-17A is produced by neutrophils (42), whereas IL-22 is biphasically produced by iNKT cells (24 h) and γδ T cells (48 h) (32). ILC3s may also produce IL-22 during *A. fumigatus* exposure (70). We have also examined mechanisms of IL-17A and IL-22 regulation during IPA, identifying such mediators as IL-1β (33), IL-23 (42), IL-7 (32), IL-21 (32), PGE2 (33), and products of 12/15-lipoxygenase signaling (31) as positive regulators and IL-15 (32) and IL-33 (33) as negative regulators. We have shown that Il1r1−/− mice (deficient in IL-1β signaling) have impaired type 17 responses during IPA (33). Likewise, inhibiting COX-2 or treating mice with IL-33 resulted in impaired type 17 responses (33). In contrast, PGE2 agonists were effective at increasing type 17 responses (33). Here, in the absence of cDC1s, we show that IL-1β and PGE2 were increased in conjunction with a decrease in IL-33, a phenotype that, based on our previous work, would promote type 17 responses. As mo-DCs (71) and an undefined CD11c+ MHC II+ DC subset (72) have been shown to produce IL-33 in the lung, the possibility exists that cDC1s regulate the type 17 axis by producing IL-33. As neutrophils were not different between WT and *Batf3*−/− mice, we hypothesize that increased type 17 responses likely function in augmenting antifungal mediator production, as we have previously reported (43). Regarding DCs, the cDC2 subset is often associated with the generation of type 17 responses (73, 74). In contrast, the absence of mo-DCs results in enhanced type 17 responses during IPA (45), suggesting that mo-DCs are negative regulators of these responses. Likewise, our data also suggest that the cDC1 subset serves to balance the magnitude of type 17 responses during IPA.

In summary, the observation that cDC1s regulate alveolar macrophage antifungal activity as well as type 17 responses is significant, as alveolar macrophages are known to be resistant to radiation therapy (75) and immunosuppressive therapy (corticosteroids and chemotherapy) (76), which are major risk factors for the development of IPA (77). Likewise, type 17 responses have been reported to be resistant to immunosuppressive therapies (78). Therefore, identifying cDC1-mediated mechanisms of alveolar macrophage and type 17 immunoregulation is of high importance, as these responses may be the only targetable effector functions to preserve during radiation or steroid-induced immunosuppression in order to minimize susceptibility to IPA. Future studies will explore specific signaling pathways involved in cDC1-AM interactions and the role of cDC1-derived mediators in modulating type 17 responses during IPA. In addition, as we are also interested in immunopathogenic mechanisms in allergic fungal asthma, future studies will explore the role of cDC1s in this model.

## SUPPLEMENTAL MATERIAL

Supplemental material mbio.02564-25-s0001.pdf Flow gating strategy and antibodies employed.

ASM does not own the copyrights to Supplemental Material that may be linked to, or accessed through, an article. The authors have granted ASM a non-exclusive, world-wide license to publish the Supplemental Material files. Please contact the corresponding author directly for reuse.
