# Supplementary material for: Type 1 conventional dendritic cells regulate innate immunity during fungal pneumonia
Source: mBio. 2025 Sep 22;16(11):e02564-25. doi: 10.1128/mbio.02564-25 (PMC12607573; doi:10.1128/mbio.02564-25)
Supplement: Supplemental material — Flow gating strategy and antibodies employed. [file mbio.02564-25-s0001.pdf]

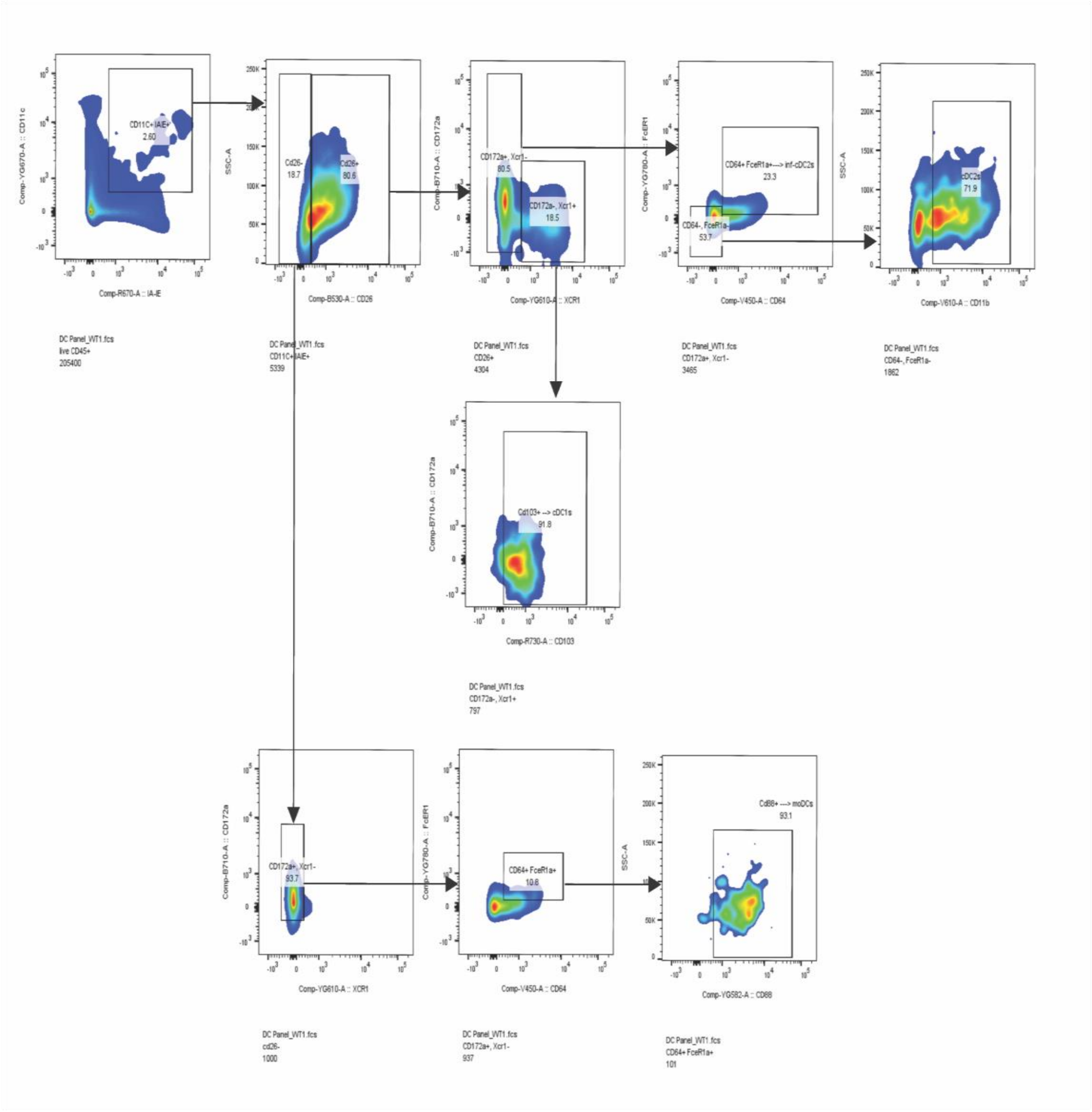

Supplemental Figure 1. Dendritic cell subset identification and gating. Lungs were collected and enzymatically digested as described in the Methods. Single-cell suspensions were stained with a viability dye followed by the antibodies in Supplemental Table 1 in the following combinations: cDC1: CD11c+ CD103+ CD26+ XCR1+ CD172a− CD45+ I-A/I-E+, cDC2: CD11c+ CD11b+ CD26+ CD64− XCR1− CD172a+ CD45+ FcεRI− I-A/I-E+, inf-cDC2: CD11c+ CD26+ CD64+ XCR1− CD172a+ FcεRI+ CD45+ I-A/I-E+ and mo-DC: CD11c+ CD26− CD64+ CD172a+ XCR1− FcεRI+ CD88+ CD45+/− I-A/I-E+.

Reagent/Resource	Source	Identifier
**anti-CD88 PE**	BioLegend	Cat #135805
**anti-I-A/I-E APC**	BioLegend	Cat #107613
**anti-CD11c PE/Cy5**	BioLegend	Cat #117316
**anti-CD11b BV605**	BioLegend	Cat #101237
**anti-CD103 AF700**	BioLegend	Cat #121441
**anti-CD172a PerCP/Cy5.5**	BioLegend	Cat #144009
**anti-CD64 BV421**	BioLegend	Cat #139309
**anti-FcεRI PE/Cy7**	BioLegend	Cat #134317
**anti-CD45 APC/Cy7**	BioLegend	Cat #103115
**anti-CD26 FITC**	BioLegend	Cat #137805
**anti-XCR1 PE Dazzle 594**	BioLegend	Cat #148233

Supplemental Table 1. Antibodies employed for dendritic cell subset identification. Lungs were collected and enzymatically digested as described in the Methods. Single-cell suspensions were stained with a viability dye followed by the antibodies listed here in the following combinations: cDC1: CD11c+ CD103+ CD26+ XCR1+ CD172a− CD45+ I-A/I-E+, cDC2: CD11c+ CD11b+ CD26+ CD64− XCR1− CD172a+ CD45+ FcεRI− I-A/I-E+, inf-cDC2: CD11c+ CD26+ CD64+ XCR1− CD172a+ FcεRI+ CD45+ I-A/I-E+ and mo-DC: CD11c+ CD26− CD64+ CD172a+ XCR1− FcεRI+ CD88+ CD45+/− I-A/I-E+.
